# Phytochemical Investigation of Polyphenols from the Aerial Parts of *Tanacetum balsamita* Used in Transylvanian Ethnobotany and Parallel Artificial Membrane Permeability Assay

**DOI:** 10.3390/plants13121652

**Published:** 2024-06-14

**Authors:** Ágnes Alberti, Eszter Riethmüller, Csenge Anna Felegyi-Tóth, Szilvia Czigle, Dóra Czégényi, Rita Filep, Nóra Papp

**Affiliations:** 1 Department of Pharmacognosy, Faculty of Pharmacy, Semmelweis University, Üllői út 26, HU-1085 Budapest, Hungary; alberti.agnes@semmelweis.hu (Á.A.); riethmuller.eszter@semmelweis.hu (E.R.); felegyi.toth.csenge.anna@semmelweis.hu (C.A.F.-T.); 2 Department of Pharmacognosy and Botany, Faculty of Pharmacy, Comenius University Bratislava, Odbojárov 10, SK-832 32 Bratislava, Slovakia; 3 Department of Hungarian Ethnography and Anthropology, University of Babeş-Bolyai of Cluj-Napoca, Horea 31, RO-400202 Cluj-Napoca, Romania; dora.czegenyi@ubbcluj.ro; 4 Department of Pharmacognosy, Faculty of Pharmacy, University of Pécs, Rókus u. 2., HU-7624 Pécs, Hungary; filep.rita@pte.hu

**Keywords:** *Tanacetum balsamita*, costmary, ethnobotany, UHPLC-MS/MS, PAMPA-BBB, PAMPA-GI, methoxyflavone

## Abstract

In this study, based on ethnobotanical data recorded in Transylvania, the polyphenolic compounds and the permeability of the aerial part’s extract of *Tanacetum balsamita* were investigated. Ultrahigh-performance liquid chromatography-tandem mass spectrometry was applied for the analysis of the extracts. Parallel artificial membrane permeability assay (PAMPA) for the gastrointestinal tract and the blood–brain barrier was conducted. In the ethanolic and aqueous extracts of the species traditionally used for wound, furuncle, and liver disorders, 92 polyphenols were characterized (e.g., flavonoid, hydroxycinnamic acid, catechin, dihydroxybenzoyl, lignan derivatives, and a monoterpene) including 54 compounds identified for the first time in the plant. In the PAMPA tests, eight components were shown to be capable of passive diffusion across the studied membranes. These include apigenin and seven methoxylated flavonoid derivatives. Based on these results, methoxylated flavonoids might promote the pharmacological potential of *T. balsamita* to be applied in the enhancement of novel remedies.

## 1. Introduction

Medicinal plants have been investigated for their traditional role since the ancient times worldwide. Traditional data inherited from generation to generation can be collected for their pivotal role for ethnobotanical and ethnomedicinal sciences, which, after comparison with those obtained from relevant sources, can provide new or underrepresented species, used parts, or indications for further analyses [1,2,3].

**Figure 1:**
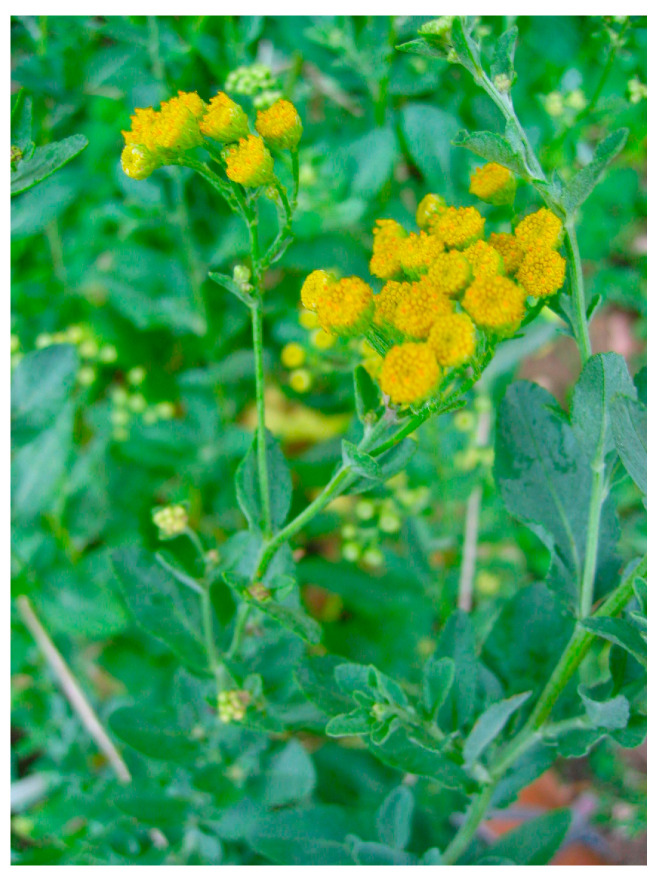
*Tanacetum balsamita* (author: Nóra Papp).

Our ethnobotanical field surveys started in 2007 in the Úz Valley in Transylvania, a part of Romania, and continued in many regions of the country. Summarizing the earlier published ethnomedicinal data from 2007 to 2019 [4,5,6,7,8], *Tanacetum balsamita* L. (costmary, Asteraceae) (Figure 1) was selected in our present work for further analyses.

*Tanacetum* genus involving species mentioned in the European, Asian, and African ethnobotany comprises various metabolites, such as terpenes, polyphenols, and polysaccharides [9,10,11,12]. The genus has a long history of use in traditional medicine, especially among Greek and early European herbalists. Based on Etymologia botanica, it comes from the Greek *tanaos* (persistent) and *athanasia* (immortality). It did not appear in Central Europe until the late 8th or early 9th century; in *Capitulare de villis vel curtis imperii* or *Repertorium Fontium*, it is known as *tanazita* in Medieval Latin [13], referring to the white flowers which retain their color and shape. The first-century Greek physician Dioscorides prescribed it as *Pyrethrum tanacetum* and *P. balsamita*, as well as *dorycnion*, *pyrinon*, *pyrothron*, and *arnopurites*; the Magi call it *purites* and the Romans *salivaris.* In this work, the root has been used against phlegm, for toothache boiled with vinegar as a mouthwash, for long-lasting chills, and paralysis [14]. In Matthioli’s works, the plant is described as *Tanacetum Athanana* [15], applied in wine, beer, vinegar, milk or honey against worms, kidney stones, as an analgesic and diaphoretic drug, for pain and swelling of the legs, and for women against enchanted infatuation as a belief [16].

In Hungarian historical works, the species has been found originally in monasteries and farms [17] and announced as Mary’s symbol in codices of the Middle Ages [18]. Before the 16th century, it was described as *Bodog anya me[n]taya* [19], while thereafter as *Boldogasszony mentája* [20], *levele*, *füve*, *tejes lapu*, *lapos menta*, *széles menta*, *biblialevél*, *bazsamint banyafű*, *bonyóvirág*, *vénasszonyvirág*, *vénasszonybűzlentyű*, *balzsammenta*, and *balzsamos aranyvirág* [21,22,23,24,25]; among them, *menta* occurs most frequently in these works.

In the early Hungarian ethnomedicinal works, the species was known as *gyönyörűséges illatú* (delicious scented) and *házi menta* (home mint) [26] and mentioned for impotency (pressed sap), inflammation (leaf), and wound (root) [27]. In gardens in the Renaissance era [28,29], it has been used for stomach disorders, fever, as an ingredient of “good spicy water” [30,31], a leaf in salads [28], for sunspots, for the pursuit of snakes [32,33,34,35], and as a component of “impairing fat” in documents of a witch-hunt from 1728 [36]. In the 19–20th century, it was mostly documented also for wound, against fleas and worms [37,38,39,40,41,42,43,44,45], as an antidote of opium, for spasm, respiratory, menstrual and stomach complaints, migraine, and epilepsy, and as an ornamental plant grown in gardens and cemeteries [46]. Under the local name *Boldogasszony tenyere*, the leaf is used for liver diseases [47,48], for helminthiasis as a tea [48], for wounds [49] and abscess as a foment [48].

In Persian ethnomedicine, it has been used for its carminative, cardiotonic, hepatoprotective, antiallergic, sedative, flavoring, and tonic effects, against migraine and dysmenorrhea [50,51,52,53,54,55,56], for diabetes [57], abscess, wound, diabetes mellitus, rheumatism and as an antipyretic agent in Turkey [12,58], for cholecystitis, dyspepsia, inflammation, and insomnia in Italy [59,60,61,62], as well as for heart and skin problems in the Hutsuls of Bucovina [63]. It is known as a spicy flavor for cosmetics, for herbal tea and various dishes in Serbia and Italy [59,60,64], and as a fumigant agent [65].

The aerial part of *T. balsamita* var. *tanacetoides* has been described in the British Pharmacopoeia (1788) for its laxative, diuretic, astringent, antiseptic, and anthelmintic effect [66]. Hoppe recommends *Flores Tanaceti* as a vermifuge and antipyretic drug in veterinary medicine, for rheumatics, for disorders of the nervous system, and as an abortive remedy [67].

The name *Tanacetum balsamita* L. was first published by Linnaeus in Species Plantarum in 1753 [68]. József Csapó lists *Tanacetum officinarum foliis bipinnatis* as the Hungarian drug name, *Tanaife* and *Tannée* as French, *Tanaceto Athanasia* as Italian, as well as *Rheinfarn* and *Wurmkraut* as German plant names [69]. The species is known actually under several synonyms, such as *Chrysanthemum balsamita* Baill., *Ch. tanacetifolium* (Desr.) Dum. Cours., *Ch. majus* Asch., *Balsamita major* Desf., *B. suaveolens* Pers.; *B. vulgaris* Willd., *B. balsamita* Rydb., *Matricaria balsamita* Desr., *Pyrethrum balsamita* Willd., *P. tanacetum* DC., *P. majus* (Desf.) Tzvelev, *Chamaemelum balsamita* E. H. L. Krause (costmary, Asteraceae) [70]. It is native to Western Asia and widely cultivated for ornamentation and locally naturalized in Europe [71,72,73]. Subspecies, varieties, and forma reported in the literature, e.g., *T. balsamita* L. ssp. *balsamitoides* (Schultz Bip.) Grierson [50,74], *T. balsamita* var. *tanacetoides* Boiss. (syn. *Ch. balsamita* var. *tanacetoides* Boiss., *Balsamita major* var. *tanacetoides* (Boiss.) Moldenke, *Pyrethrum balsamita* var. *tanacetoides* Boiss.), *T. balsamita* var. *balsamita* [70] as a perennial species native to Western Asia [66,75], *Ch. balsamita* f. *tanacetoides* (Boiss.) B. Boivin, and *Balsamita major* var. *major*, *B. major* ssp. *major* [70].

The aerial part of *T. balsamita* contains essential oil components, e.g., mono- and sesquiterpenes [52,74,75,76,77,78,79,80,81,82,83,84,85,86]. Based on these components, carvone, camphor, camphor/thujone and carvone/α-thujone chemotypes have been described [79,87]. The herb contains, furthermore, diterpenes [88], flavonols [52,83,89,90,91,92,93,94,95,96], flavones, e.g., diosmetin and acacetin derivatives, as well as phenolic acids [90,91,92,94,96,97], coumarins, and steroids [86].

The antioxidant activity of costmary was studied by the 2,2′-azinobis(3-ethylbenzothiazoline-6-sulfonic acid (ABTS), cupric reducing antioxidant capacity (CUPRAC), 2,2-diphenyl-1-picrylhydrazyl (DPPH), and ferric reducing antioxidant power (FRAP) assays [86,90,94,98,99]. The antimicrobial activity was tested in the case of the aerial part and the extracted essential oil by different methods [77,81,85,86,92,99,100,101]. The plant was studied for cytotoxic activity on human embryonal skin fibroblast cells using the MTT cytotoxicity assay [81], on Monkey kidney cell lines [101], and on THP-1 monocyte leukemic cells [94]. Studies reported its insecticidal [102], analgesic [83], and anti-inflammatory effect [83,103,104], as well as the inhibitory potential for smooth muscle contraction of ruminants [93], and for tyrosinase and α-amylase activity [94], whilst an increasing effect for the activity of intestinal enzymes in common carp [105].

The aim of this study was to sum ethnobotanical data on *T. balsamita* collected in selected regions in Transylvania, Romania. For the identification of polyphenols, the traditionally used aerial part of the plant was studied by ultrahigh-performance liquid chromatography-diode-array detection-electrospray ionization mass spectrometry (UHPLC-DAD-ESI-MS), and the effective permeability of the components in the plant extracts by the parallel artificial membrane permeability assay for the gastrointestinal tract (PAMPA-GI) and the blood–brain barrier (PAMPA-BBB).

## 2. Results

### 2.1. Ethnobotanical Data in Selected Areas in Transylvania

The plant is described as *vénasszonvirág* and *vénasszonyvirág* (= old women’s flower) along the Nagy-Homoród, while *bódogasszonlapi* and *boldogasszonylapi* (= glad/blessed women’s leaf) in Ghimeş and Úz Valley. The origin of the name *vénasszonyvirág* was defined: “…ami elég szívós, s nincs sok baj vele, s hosszan virágzik”. (= …which is hard/wiry, it does not cause problems, beeing in flower for a long time). People mentioned its use as an ornamental plant was known for a long time in the studied regions: “Minden háznál vót ezelőtt ilyen virág”. (= This plant has been found in all homegardens long ago). In addition, the aerial part was mentioned for wound and liver diseases in the Ghimeş region and for furuncle in the Úz Valley as a decoction. Along the Nagy-Homoród, old women put the fragrant leaves of the plant into the Bible or prayer books during the mass as a refreshing material.

### 2.2. Qualitative Analysis of Phenolic Compounds with UHPLC-DAD-ESI-MS/MS

**Figure 2:**
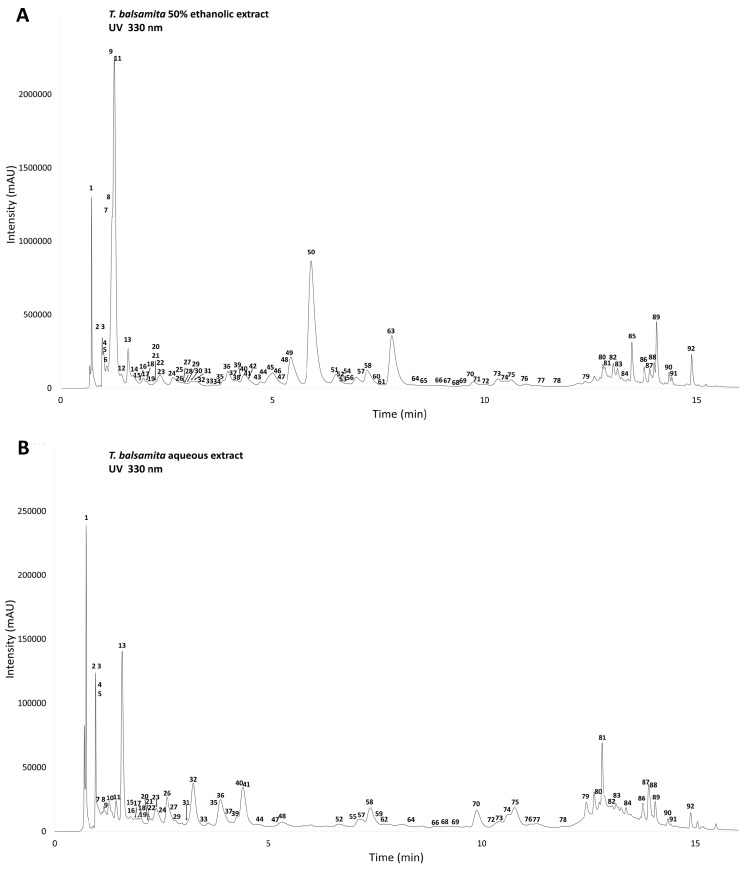
UHPLC-DAD chromatograms of the *T. balsamita* herb extracts (recorded at 330 nm); (**A**): 50% (*v*/*v*) ethanol extract; (**B**): aqueous extract. Compound numbers refer to Table 1.

Ultrahigh-performance liquid chromatography coupled to diode-array detection and electrospray ionization tandem mass spectrometry (UHPLC-DAD-ESI-MS/MS) in negative ionization mode was used to characterize the phenolic profile of the *T. balsamita* ethanolic and aqueous extracts. UHPLC-DAD chromatograms of the 50% (*v*/*v*) ethanol and aqueous extracts (recorded at 330 nm) are presented in Figure 2A and Figure 2B, respectively. A total of 92 compounds were characterized by comparing their retention times, UV spectra, deprotonated molecular ions, and fragment ions generated in their collision-induced dissociation with the literature data; results are shown in Table 1.

The extractable matter content was higher in the hydroethanolic extract (as shown by the remarkably higher peak intensities) and its composition was also greatly different. Caffeoylquinic acids (compounds **9** and **11**) and dicaffeoylquinic acids (**49**, **50**, **68**) were the predominant constituents, while flavonoid methyl ethers (**85**, **89**, **92**) also prevailed. In the aqueous extract, the more polar caffeoyl-*O*-hexoside (**2**), dihydroxybenzoyl-*O*-hexoside (**3**), apigenin-6,8-di-*C*-hexoside (**13**) as well as an apigenin- and some methoxyflavone-*O*-diglycosides with hexuronic acid moieties (**36** and **32**, **40**, **41**) were abundant. Methoxyflavone-*O*-acylglycosides (**70**, **75**, **81**) and flavonoid methyl ethers (**87**, **88**) were representative apolar constituents in the aqueous extract.

#### 2.2.1. Flavonoid Derivatives

In accordance with a recent study [94], mainly flavone-*O*- and *C*-glycosides were detected in *T. balsamita* extracts, besides flavonol and chalcone derivatives. During the collision-induced dissociation of flavonoid-*O*-glycosides, fragment ions corresponding to the deprotonated apigenin, luteolin, and quercetin aglycones generated by the loss of the sugar units were observed at *m/z* 269, 285, and 301, respectively. Neutral losses deriving from the cleavage of a pentose (132 Da), a hexose (162 Da), a hexuronic acid 176 Da), an acetylhexose (204 Da), or an acetylhexuronose (218 Da) moiety were also characteristic [143]. In case of 3-*O*-glycosylated quercetin derivatives, the homolytic cleavage of the saccharide moiety resulted in the formation of the [Y_0_−H]^−•^ radical product ion at *m/z* 300 [134]. Based on these, compounds **35**, **42**, **36**, and **52** detected at *m/z* 609, 463, 607, and 445 were tentatively identified as rutin (quercetin-3-*O*-rhamnosyl-glucoside), quercetin-3-*O*-hexoside, apigenin-*O*-hexuronosyl-hexoside, and apigenin-*O*-hexuronoside. Similarly, **37** (*m/z* 461), **67** (*m/z* 417), **59**, and **61** (both *m/z* 503) were characterized as luteolin-*O*-hexuronoside, luteolin-*O*-pentoside, and luteolin-*O*-acetylhexuronoside isomers, respectively [12,94,135,139].

Compounds **15**, **23**, **38** with pseudomolecular ions at *m/z* 637, 623, and 593 presented the aglycone anion at *m/z* 285 and further fragment ions at *m/z* 461, 447, and 417, respectively. Accordingly, the following neutral losses were observed: 176 + 176, 176 + 162, and 176 + 132; thus, **15**, **23**, and **38** were assigned as luteolin-di-*O*-hexuronoside, luteolin-*O*-hexuronosyl-hexoside, and luteolin-*O*-hexuronosyl-pentoside, respectively. Analogously, **44** was characterized as luteolin-*O*-hexuronosyl-acetylhexoside [12,94,118,128,138,139].

Neutral losses of 162, 146, and 176 Da can also point to the cleavage of caffeoyl, coumaroyl, or feruloyl moieties, respectively. However, these hydroxycinnamic acids esterifying flavonoid-glycosides also exhibit characteristic fragment ions contributing to their identification. Fragment ions corresponding to the deprotonated hydroxycinnamic acids were detected at *m/z* 179, 163, and 193 for caffeic acid, coumaric acid, and ferulic acid, respectively. Ions deriving from the additional cleavage of a H_2_O molecule were also present at *m/z* 161 for caffeic acid, *m/z* 145 for coumaric acid, and *m/z* 205 for sinapic acid. The cleavage of the acylated saccharide moiety, i.e., coumaroylhexose, caffeoylhexose, caffeoylhexuronose, and sinapoylhexose resulted in neutral losses of 308, 324, 338, and 368 Da, respectively [143]. Compounds **57**, **76**, and **74** presented a fragment ion at *m/z* 145 arising from the neutral loss of 146 Da; thus, these compounds were assumed to be coumaroylhexoside derivatives of luteolin and apigenin. Compounds **28** and **43** showing a neutral loss of 162 Da and a caffeoyl (caffeic acid−H_2_O) fragment ion at *m/z* 161 were presumed as luteolin-*O*-hexuronosyl-caffeoylhexuronoside and luteolin-*O*-hexuronosyl-caffeoylhexoside, respectively [113].

Methoxyflavonoids (**82**, **83**, **85**, **87–92**), their glycosides (**31**, **32**, **39–41**, **45**, **47**, **48**, **55**, **58**, **60**, **69**, **77**, **78**), and acylglycosides (**30**, **46**, **48**, **53**, **54**, **64**, **70**, **75**, **80**) have also been detected in *T. balsamita* extracts. In case of these compounds, the cleavage of a methyl radical (15 Da) leading to the formation of [M−CH_3_]^−•^ ions points to the presence of a methoxy group [150,151]. Compounds **88**, **90**, **91** as well as **92** suffered 3 neutral losses of 15 Da (*m/z* 359 → 344 → 329 → 314 and *m/z* 343 → 328 → 313 → 298) indicating a trihydroxy-trimethoxyflavone (**88**, **90**, **91**) or dihydroxy-trimethoxyflavone (**92**) structure. In the mass spectra of **83**, **85**, and **89**, two neutral losses of 15 Da occurred (*m/z* 345 → 330 → 315 and *m/z* 329 → 314 → 299) suggesting a tetrahydroxy-dimethoxyflavone (**83**, **85**) or a trihydroxy-dimethoxyflavone (**89**) skeleton. Compounds **82** and **87** with a single loss of 15 Da were characterized as tetrahydroxy-methoxyflavone and trihydroxy-methoxyflavone, respectively [94,140]. The structures of glycoside and acylglycoside derivatives of methoxy-flavone were proposed as detailed previously. The neutral losses yielded by the cleavage of sugar residues and cinnamoyl moieties, together with the fragment ions corresponding to the deprotonated and/or dehydrated cinnamic acids were analyzed for the structural characterization of flavonoid methyl ether derivatives [113,129,137,143].

During the analysis of apigenin-*C*-glycosides, the neutral losses of 120 and 90 Da for *C*-hexoses, as well as 90 and 60 Da for *C*-pentoses, were characteristic [152]. Compound **13** presented the [M−H]^−^ ion at *m/z* 593 and its fragment ions at *m/z* 503, 473, 383, and 353, indicating two neutral losses of 120 Da (*m/z* 593 → 473 → 353); thus, it was identified as apigenin-6,8-di-*C*-hexoside [94,110,116,117]. Compound 33 showing a neutral loss of 120 Da (*m/z* 431 → 311) did not exhibit a [M−H−2H_2_O]^−^ fragment ion (at *m/z* 395) typical for flavone-6-*C*-hexosides; thus, it was suggested to be apigenin-8-*C*-hexoside [110,131,132]. Compound **21** presenting a neutral loss of 120 Da and an abundant [M−H−60]^−^ ion (at *m/z* 503), characteristic of flavone-6-*C*-pentosides but not of flavone-8-*C*-pentosides, was proposed to be apigenin-8-*C*-hexosyl-6-*C*-pentoside [117,122,123].

Furthermore, dihydrochalcone-*C*-glycosides (**66**, **68**) were also tentatively characterized, by comparing their MS spectra with the literature [112,113].

#### 2.2.2. Hydroxycinnamic Acid Derivatives

Characteristic fragment ions of caffeic, coumaric, ferulic, and dihydrosinapic acid derivatives (at *m/z* 179–161, 163–145, 193–175, and 225–207, respectively) were used for their tentative characterization, as described earlier. The neutral losses of 162 and 176 Da referred to a hexose and a hexuronose moiety; therefore, compounds **2**, **12**, **24**, **72, 8**, and **16** were identified as caffeoyl-*O*-hexoside (**2**), two *p*-coumaroyl-*O*-hexoside isomers (**12**, **24**), coumaroyl-caffeoyl-*O*-hexoside (**72**), dihydrosinapoyl-*O*-hexuronosyl-hexoside (**8**), and dihydrosinapoyl-*O*-hexoside (**16**) [108,113,124].

Compounds **4**, **9**, **11**, and **14** were characterized as caffeoylquinic acid isomers, while **22** and **26** were proposed to be feruloylquinic acids. The isomers could be differentiated based on the relative intensities of their fragment ions. In case of **4** and **26**, the abundance of the *m/z* 191 (deprotonated quinic acid) fragment ion and the *m/z* 179 (deprotonated caffeic acid) or the *m/z* 193 (deprotonated ferulic acid) secondary fragment ions referred to 3-*O*-caffeoylquinic acid and 3-*O*-feruloylquinic acid, respectively. Compounds **9**, **14**, and **22**, presenting the base peak at *m/z* 191 without any secondary peaks, were identified as 5-*O*-caffeoylquinic acid isomers and 5-*O*-feruloylquinic acid. According to Jaiswal et al., the *cis* isomer of cinnamoylquinic acids is assumed to be the more hydrophobic and is therefore eluted at higher retention times. Thus, **9** was characterized as *trans*-5-*O*-caffeoylquinic acid, while (**14**) as *cis*-5-*O*-caffeoylquinic acid. The presence of the fragment ion at *m/z* 173 indicated a 4-substituted isomer; therefore, **11** was characterized as 4-*O*-caffeoylquinic acid [108,111,127,133].

Similarly, dicaffeoylquinic acid isomers with [M−H]^−^ ions at *m/z* 515 could also be distinguished based on their fragment ions. In case of 3,4-*O*-dicaffeoylquinic acid (**49**), the fragment ion at *m/z* 173 was the base peak; however, the intensity of the secondary peaks at *m/z* 191 and 179 was relatively high. The domination of the fragment ion at *m/z* 191 indicated the 3,5-*O*-dicaffeoylquinic acid isomer for **50**, while the base peak at *m/z* 173 with relatively low intensity secondary peaks referred to the 4,5-*O*-dicaffeoylquinic acid isomer (**63**). Compound **51** presenting [M−H−H_2_O]^−^, [quinic acid−H]^−^ and [caffeic acid−H]^−^ at *m/z* 335, 191, and 179, respectively, pointed to the 1,3-*O*-dicaffeoylquinic acid isomer [109,133,135,141,142]. Additionally, a tri-*O*-caffeoylquinic acid isomer (**84**) was also identified in the extracts [111,133,148,149].

Further caffeoyl derivatives with distinctive fragment ions have also been detected. Compounds **6**, **17**, **71**, and **25** with the following pseudomolecular and fragment ions: *m/z* 357 → 195, *m/z* 339 → 179, *m/z* 461 → 323, 299, and *m/z* 343 → 167 were assigned as caffeoyl-*O*-pentahydroxyhexanoic acid (caffeoyl-*O*-gluconic acid), caffeoyl-*O*-dimethyl-dihydroxybutanedioic acid, salicylic acid caffeoyl-*O*-hexoside, and vanillyl-*O*-hexuronoside, respectively [113,125,126,135].

#### 2.2.3. Other Constituents

The fragmentation patterns of compounds **3** and **5** indicated the presence of a dihydroxybenzoic acid moiety with its typical fragment ion at *m/z* 153. The neutral losses of 162 Da and 2 × 132 Da referred to a hexose and two pentose moieties; thus, the compounds were characterized as dihydroxybenzoyl-*O*-hexoside and dihydroxybenzoyl-di-*O*-pentoside [94,109,112,113].

Compounds **7** and **10** showing the characteristic ions at *m/z* 305 and 225, as well as *m/z* 307 and 227 were supposed to be an epigallocatechin/gallocatechin isomer and a hydrated catechin/epicatechin isomer, respectively [114,115].

Compound **18** (*m/z* 535) was identified as hydroxypinoresinol-*O*-hexoside. The presence of another lignan was presumed for **73**: medioresinol-*O*-hexoside or eucommin A. However, according to the literature, the dihydrosinapoyl-caffeoyl-*O*-hexoside structure presenting the same fragment ions may also be probable for **73** [109,113,144].

Compound **19** presented a pseudomolecular ion at *m*/*z* 389 and fragment ions at *m*/*z* 345, 227, and 209 indicating the loss of a CO (*m*/*z* 389 → 345) and a hexose molecule (*m*/*z* 389 → 209). Based on the literature, these characteristics suggested the structure of oleoside/secologanside [120,121].

### 2.3. Parallel Artificial Membrane Permeability Assay (PAMPA)

**Figure 3:**
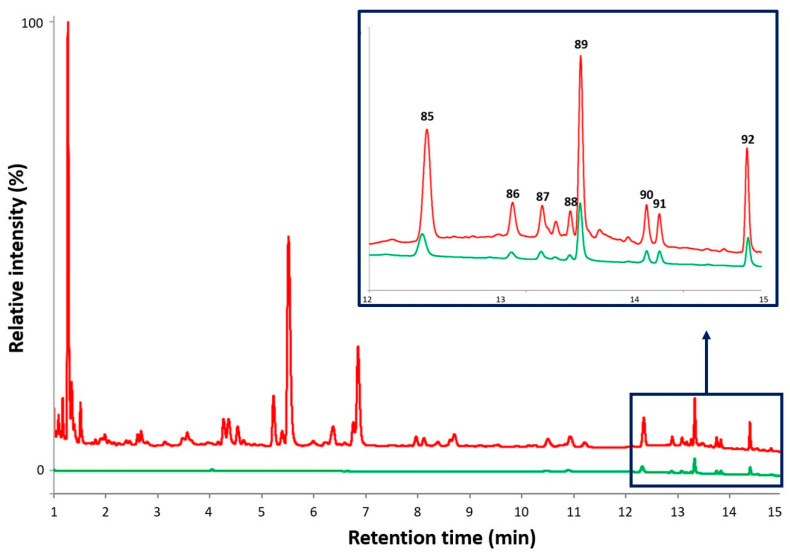
Representative UHPLC-DAD chromatograms (max plot) of the PAMPA-BBB studies: *T. balsamita* 50% (*v*/*v*) ethanolic extract (TbE) before (red) and acceptor solution after the PAMPA-BBB experiment (green).

The ability of the compounds in *T. balsamita* extract (TbE and TbW) to cross biological membranes of the gastrointestinal (GI) tract and the blood–brain barrier (BBB) by passive diffusion was investigated using the PAMPA model [153]. The coupling of this assay with UHPLC separation allowed the rapid simultaneous investigation of the membrane permeability of the compounds present in the extract (Figure 3). The PAMPA is considered to be one of the most effective and versatile screening tools for early drug discovery. Due to the artificial nature of the membrane used in the assay, only passive transport mechanisms can occur, unlike in cell-based assays. This is particularly important in the case of plant extracts due to their complexity, as the evaluation of results obtained by different co-existing mechanisms (e.g., active transport, or metabolism) can be challenging. Furthermore, no significant differences in effective permeabilities are observed in the PAMPA, whether assessed with single compounds or mixtures [153].

In the case of both extracts (TbE and TbW), eight compounds (**85**–**92**) were detected in the acceptor phase of both PAMPA models. Of these, apigenin (**86**) was identified by comparison of its chromatographic, as well as UV and mass spectrometric behavior to the authentic standard. The remaining seven methoxylated flavonoid derivatives (**85, 87**–**92**) were characterized by quadrupole time-of-flight mass spectrometry (QTOF-MS).

Based on the chromatographic peak areas (UHPLC-DAD), these compounds were present in higher relative concentrations in the extract prepared with 50% (*v*/*v*) ethanol (TbE); therefore, data from this extract were used for the calculation of the log*P_e_* values (n = 9), as the larger peak areas help to obtain more accurate results, especially in the case of minor constituents. All of these eight flavonoids exhibited log*P_e_* values greater than −6.0 in the PAMPA-BBB studies and greater than −5.0 in the PAMPA-GI experiments (Table 2).

Accordingly, these components can be considered as having good membrane permeability [153]; thus, it can be assumed that they are absorbed in the gastrointestinal tract and cross the blood–brain barrier by passive diffusion. Although, it must be pointed out that due to the artificial nature of the membrane used in the assays, merely passive transport mechanisms can occur, and active (e.g., efflux) transport of the compounds cannot be studied.

Nevertheless, it is also worth noting that apigenin and the methoxylated flavonoids axillarin, sudachitin, casticin, and nevadensin isolated from a related species, *Tanacetum parthenium* (L.) Sch. Bip., have already been reported to have good permeability in the PAMPA-BBB model [140].

## 3. Discussion

*Tanacetum balsamita* has been known for centuries in European ethnomedicine and as an ornamental plant. Among the recorded use of the species in the study areas, treatment for wounds was also documented earlier in Transylvania [27,48,49,154], Bucovina [63], and Turkey [12,58], for liver diseases also in Transylvania [47,48] and in Persian traditional medicine [52], while similar to our record as a refreshment, it was found in tonic and flavoring preparations in Iran [52], Lithuania [90], Serbia, and Italy [59,60,64]. In the ethnodermatological aspect, our record to treat furuncle was documented as a decoction in Úz Valley.

In search of the potentially bioactive constituents, we tentatively characterized 91 phenolic compounds and a monoterpene in *T. balsamita* extracts by UHPLC-ESI-MS. In line with the literature data, the most prevalent constituents were flavonoids and cinnamoylquinic acids, with a prominent difference between the composition of the aqueous and the 50% ethanolic extract. The former mainly comprised apigenin-*C*-glycosides, methoxyflavone-di-*O*-glycosides, and methoxyflavone-*O*-acylglycosides, while caffeoylquinic acids and methoxyflavone aglycones were prevailing in the latter.

Benedec et al. identified the flavonols kaempferol and quercetin as well as rutin, isoquercitrin, and quercitrin (the glycosides of quercetin) in two *T. balsamita* varieties [97]. We also detected flavonol-*O*-glycosides such as rutin (**35**) and quercetin-3-*O*-hexoside (**42**) as minor constituents; however, kaempferol, quercetin, and quercitrin (quercetin-3-*O*-rhamnoside) were not present in our extracts.

Bączek et al. concluded that cichoric acid is the dominant phenolic compound, and apigenin-7-*O*-glucoside is the main flavonoid of *T. balsamita*. They also determined the quantities of caffeic acid, 3-*O*-caffeoylquinic acid, rosmarinic acid, and the flavonoids quercetin, luteolin-7-*O*-glucoside, and chrysoeriol [99]. Surprisingly, we did not detect cichoric acid, caffeic acid, and rosmarinic acid. Additionally, we only observed *C*-glycosides of apigenin (**21**, **33**) as well as hexurunosides (**37**, **59**, **61**), a pentoside (**67**), di- (**15**, **23**, **38**), and acylglycosides (**28**, **43**, **44**, **57**), but no glucoside for luteolin. We described apigenin-8-*C*-hexoside (**33**) and apigenin-8-*C*-hexosyl-6-*C*-pentoside (**21**) in costmary for the first time.

In alignment with the results of Pukalskas et al. who identified the major antioxidative components of costmary, we also detected 5-*O*-caffeoylquinic acids (**9**, **14**), 3,5-*O*-dicaffeoylquinic acid (**50**), trihydroxy-dimethoxyflavone (**89**), and tetrahydroxy-dimethoxyflavon (**83**, **85**) [90]. We reported the presence of distinct *trans* (**9**) and *cis* (**14**) isomers of 5-*O*-caffeoylquinic acid in extracts of this plant for the first time.

The lipophilic and polar flavonoids of *T. balsamita* were analyzed in previous studies [94,155]. In agreement with these results, we also identified a trihydroxy-methoxyflavone (**87**), presumably hispiduline; a dihydroxy-methoxyflavone (**82**), nepetin or isorhamnetin; a trihydroxy-dimethoxyflavone (**89**), jaceosidin or cirsiliol; two tetrahydroxy-dimethoxyflavones (**83**, **85**), probably spinacetin and axillarin, and a dihydroxy-trimethoxyflavone (**92**), eupatilin or santin. In a recent paper, two quercetagetin-trimethyl ether (trihydroxy-trimethoxyflavone) isomers were detected in *T. balsamita* [94]. However, in our work, we report three trihydroxy-trimethoxyflavone isomers (**87**, **90**, **92**) for the first time. After comparing the retention times of the compounds with the literature data, **90** can be presumed as the new metabolite.

In addition, the flavan-3-ols epigallocatechin/gallocatechin isomer (**7**) and catechin/epicatechin hydrated (**10**), the secoiridoid glycoside oleoside or secologanside (**19**), the lignans hydroxypinoresinol-*O*-hexoside (**18**), and medioresinol-*O*-hexoside or eucommin A (**73**), as well as vanillyl-*O*-hexuronoside (**25**), have been proposed in *T. balsamita* for the first time.

Besides the phytochemical analyses, we performed PAMPA experiments to assess the capability of the constituents in costmary extracts to cross biological membranes by passive diffusion. According to our results, lipophilic flavonoids are presumably absorbed in the gastrointestinal tract and cross the blood–brain barrier by passive diffusion. Thus, these may contribute to the biological effects of *T. balsamita* such as the in vivo hepatoprotective activity observed in rats [156], or the sedative [54], and anti-migraine actions [157] reported in ethnobotanical studies.

According to the literature data, *T. balsamita* extracts characterized by polyphenols such as flavonol glycosides and hydroxycinnamic acid derivatives exerted in vitro antioxidant effects [97,99,158]. Extracts of costmary and other *Tanacetum* species containing caffeoylquinic acids and flavonoids also showed fungistatic and antibacterial effects [99,159,160]. However, the results of these in vitro experiments should be assessed carefully. Most of the constituents presumed to be responsible for the antioxidative and antibacterial effects could not permeate through the membrane in our PAMPA studies and, thus, might not be absorbed in the gastrointestinal tract.

## 4. Materials and Methods

### 4.1. Ethnobotanical Survey and Research Areas

The studied settlements and data were selected based on earlier surveys conducted in the period of 2007–2019 in Transylvania, Romania. Lunca de Jos (32 informants = IF/1091 inhabitants = IH) in the Ghimeş region (Bacău County) and Cinod (45 IF/200 IH) and Egershec (25 IF/100 IH) in the Úz Valley (Harghita County) are inhabited by the Csángó people, while Călugăreni (15 IF/52 IH), Ghipeș (12 IF/138 IH), Locodeni (10 IF/83 IH), and Petreni (12 IF/120 IH) along the Nagy-Homoród river are inhabited by the Székely people (Harghita County). The Hungarian language skills of rural people facilitated the process of semi-structured interviews, complemented with the explanations of local dialects in all villages. Except for Lunca de Jos, villages are not provided by permanent pharmaceutical, medical, and veterinary services. People work mostly in agriculture and livestock farming in a close relationship with nature, involving plants’ use from wild habitat or cultivation based on their own experiences and observation. In this study, only the data mentioned on *T. balsamita* are summarized. The visited informants aged between 62 and 85 years were asked for the vernacular name, cultivation, preparation, and use of the plant. The original quotations were written in *italics* between inverted commas according to the folk terminology of Csángós and Székelys.

### 4.2. Plant Material and Sample Preparation

Aerial parts of *T. balsamita* were collected in the EGSC-Melius Medicinal Plant Garden, Pécs, Hungary, in June 2022. The herb was dried at room temperature and stored in the dark until analyses. The voucher specimen of the species labelled with a unique code was deposited at the Department of Pharmacognosy, University of Pécs, Pécs, Hungary (Voucher code: TB_06). The plant name follows the terminology of The World Flora Online (WFO, 2023) [70].

### 4.3. Reagent and Chemicals

Ethanol, as well as HPLC grade methanol and acetonitrile, was purchased from Molar Chemicals Kft. (Halásztelek, Hungary). Acetic acid 100% for HPLC LiChropur™ was acquired from Sigma-Aldrich (Steinheim, Germany). Dimethyl sulfoxide (DMSO), *n*-dodecane, hydrochloric acid (HCl), sodium hydroxide (NaOH), disodium hydrogen phosphate heptahydrate (Na_2_HPO_4_·7H_2_O), and sodium dihydrogen phosphate monohydrate (NaH_2_PO_4_·H_2_O) were obtained from Reanal-Ker (Budapest, Hungary), while apigenin, caffeine and rutin standards, phosphatidylcholine, cholesterol, the porcine polar brain lipid extract, and the PBS tablet (Phosphate Buffered Saline, pH 7.4) were purchased from Merck (Darmstadt, Germany). High-purity water was gained by a Millipore Direct Q5 Water Purification System (Billerica, MA, USA).

For UHPLC-MS and PAMPA analyses, the aqueous and 50% (*v*/*v*) ethanolic extracts were obtained by extracting 3.0 g of herb powder in 30 mL of distilled water (TbW) or 50% (*v*/*v*) ethanol (TbE) using an ultrasonic bath (three times, 30 min each) at room temperature. The extracts were distilled to dryness under reduced pressure with a rotary evaporator (Büchi Rotavapor R-200, Flawil, Switzerland) at 45 °C. The residues were dissolved in 20 mL of 70% (*v*/*v*) HPLC grade methanol and filtered through Minisart RC 15 0.2 µm syringe filters (Sartorius AG, Goettingen, Germany).

### 4.4. Phytochemical Analyses by Ultrahigh-Performance Liquid Chromatography (UHPLC) Coupled to Diode-Array Detector (DAD) and Mass Spectrometry (MS)

#### 4.4.1. UHPLC Conditions

For the analysis of the TbW and TbE extracts and the samples from the PAMPA studies, an ultrahigh-performance liquid chromatography-diode-array detection-mass spectrometry (UHPLC-DAD-MS) method was developed. Briefly, an ACQUITY UPLC H-Class PLUS System (Waters Corporation, Milford, MA, USA) hyphenated with a quaternary solvent delivery pump (QSM), an auto-sampler manager (FTN), a column compartment (CM), and a photodiode array (PDA) detector (Waters Corporation, Milford, MA, USA) were employed. The chromatographic separation was performed using an Acquity UPLC BEH C18 (Waters, Dublin, Ireland) (100 mm × 2.1 mm i.d., 1.7 µm) column, with column temperature: 30 °C. The mobile phase consisted of 0.1% formic acid in water (eluent A) and acetonitrile (eluent B). All aqueous solvents were filtered through MF-Millipore (Millipore, Billerica, MA, USA) (0.45 µm, mixed cellulose esters) membrane filters. The following gradient elution was applied at a flow rate of 0.3 mL/min: 0 min 15.0% B, 10.0 min 25.0% B, 16.0 min 80.0% B, 16.5 min 100.0% B, 19.0 min 100.0% B, 19.5 min 15.0% B. UV spectra and chromatograms were recorded at 200−400 nm. The injection volume was 5 µL.

#### 4.4.2. MS Conditions

Mass spectrometric analyses were performed with a Xevo Q-TOF instrument equipped with an electrospray ionization source (ESI) (Waters Corporation). ESI conditions were as follows: capillary voltage 2.6 kV, sampling cone voltage 40 V, source temperature 120 °C, desolvation temperature 300 °C, desolvation N_2_ gas flow 600 L/h. High purity nitrogen was used as collision gas, and the collision energy was changed between 10 eV and 45 eV, depending on the analyzed structure. Full-scan mass spectra were acquired over the range of *m/z* 100–2000 in negative ionization mode. The Masslynx 4.1 software was used for data acquisition and qualitative analysis.

### 4.5. Parallel Artificial Membrane Permeability Assay (PAMPA)

A parallel artificial membrane permeability assay (PAMPA) was used to determine the effective permeability (*Pe*) for the components of *Tanacetum* extracts prepared with water (TbW) and 50% (*v*/*v*) aqueous ethanol (TbE). Stock solutions of the extracts (100 mg/mL in DMSO) were diluted with the defined buffer (pH 7.4 for the PAMPA-BBB and pH 6.8 for the PAMPA-GI assays) to obtain the donor solutions (composition: 594.0 μL buffer + 6.0 μL stock solution). The buffers were prepared as follows: pH 6.8: 20.2 g Na_2_HPO_4_·7H_2_O and 3.4 g NaH_2_PO_4_·H_2_O dissolved in distilled water to achieve the final volume of 1000.0 mL, pH adjustment with 0.5 M NaOH or 0.5 M HCl; pH 7.4: one PBS tablet (Phosphate Buffered Saline, pH 7.4; Sigma Aldrich) dissolved in 200.0 mL distilled water. Donor solutions were filtered through Phenex-RC 15 mm, 0.2 μm syringe filters (Gen-Lab Ltd., Budapest, Hungary).

For the PAMPA-BBB test, 5 μL of porcine polar brain lipid extract (PBLE) solution (16.0 mg PBLE + 8.0 mg cholesterol dissolved in 600.0 μL *n*-dodecane) was applied for each well of the 96-well polycarbonate-based filter donor plates (top plate) (Multiscreen™-IP, MAIPN4510, pore size 0.45 μm; Merck). For the PAMPA-GI assay, the wells of the top plate were coated with 5 μL of the mixture of 16.0 mg phosphatidylcholine and 8.0 mg cholesterol dissolved in 600.0 μL *n*-dodecane. Some 150.0 μL aliquots of the filtrated donor solutions were placed on the membrane. The 96-well PTFE acceptor plates (bottom plates) (Multiscreen Acceptor Plate, MSSACCEPTOR; Merck) were filled with 300.0 μL buffer solution (0.01 M PBS buffer, pH 7.4). The donor plate was placed upon the acceptor plate, and both plates were incubated together at 37 °C for 4 h in a Heidolph Titramax 1000 Vibrating platform shaker (Heidolph, Schwabach, Germany).

After incubation, sandwich plates were separated, and the concentrations of each compound in the starting donor solution and in the acceptor and donor wells were determined in triplicate by chromatographic peak areas derived from the UHPLC-DAD method described above. UV spectra and chromatograms were recorded at 200–400 nm, and the chromatograms acquired at the UV absorption maxima of each compound were used for data evaluation. The effective permeability and the membrane retention in the PAMPA-BBB and the PAMPA GI experiments were calculated using data from the chromatograms of the TbE extract by Equations (1)–(4), respectively [161]:

(1) $$P_{e}=\frac{-2.303}{A(t-\tau_{SS})}\cdot \left(\frac{V_{A}\cdot V_{D}}{V_{A}+V_{D}}\right)\cdot lg\left[1-\left(\frac{V_{A}+V_{D}}{\left(1-MR\right)\cdot V_{D}}\right)\times \left(\frac{C_{A}\left(t\right)}{C_{D}\left(0\right)}\right)\right]$$

(2) $$P_{e}=\frac{-2.303\cdot V_{D}}{A(t-\tau_{SS})}\cdot \left(\frac{1}{1+r_{a}}\right)\cdot lg\left[-r_{a}+\left(\frac{1+r_{a}}{1-MR}\right)\times \left(\frac{C_{D}\left(t\right)}{C_{D}\left(0\right)}\right)\right]$$

where *Pe* is the effective permeability coefficient (cm/s), *A* is the filter area (0.24 cm^2^), *V_D_* and *V_A_* are the volumes in the donor (0.15 cm^3^) and acceptor phases (0.30 cm^3^), *t* is the incubation time (s), *τ_S__S_* is the time (s) to reach steady-state (240 s), *C_D_*(*t*) is the concentration (mol/cm^3^) of the compound in the donor phase at time *t*, and *C_D_*(0) is the concentration (mol/cm^3^) of the compound in the donor phase at time 0. MR is the estimated membrane retention factor (the estimated mole fraction of solute lost to the membrane), and *r_a_* is the sink asymmetry ratio (gradient-pH-induced), defined as follows:

(3) $$r_{a}=\frac{V_{D}}{V_{A}}\times \frac{P_{e}^{\left(A\to D\right)}}{P_{e}^{\left(D\to A\right)}}$$

(4) $$MR=1-\frac{C_{D}\left(t\right)}{C_{D}\left(0\right)}-\frac{V_{A}}{V_{D}}\frac{C_{A}\left(t\right)}{C_{D}\left(0\right)}$$

All experiments were performed in three triplicates on three consecutive days (n = 9); caffeine standard was used as positive, while rutin as negative control.

## 5. Conclusions

Ethnomedicinal surveys are of pivotal importance to document and maintain traditional treatments by plants in Transylvania, Romania, and to select species for further analyses. The selected *Tanacetum balsamita* is applied in recent ethnomedicine in the studied regions in Transylvania nowadays. In our work, polyphenolic compounds of *T. balsamita* were detected by UHPLC-MS/MS, including 54 constituents identified for the first time in the plant. The PAMPA study of the plant extracts revealed eight compounds with good permeability across the membranes of the gastrointestinal tract and the blood–brain barrier. Based on our recent results and previous data, it can be assumed that methoxylated flavonoids contribute to the pharmacological activity of *Tanacetum* species. Therefore, further studies investigating the structure, biological activity, and pharmacokinetic properties of *T. balsamita* flavonoids would be of great interest.

## Figures and Tables

**Table 1:** LC-MS/MS data and tentative characterization of compounds from the herb of *Tanacetum balsamita*.

No.	t_R_ (min) ^a^	[M−H]^−^ (*m/z*)	Fragment Ions (*m/z*)	Tentative Characterization	Presence of Compounds		Reference
					TbE ^b^	TbW ^b^	
**1**	0.75	133	115, 89, 73, 71	malic acid ^d.^	+	+	[106,107]
**2**	0.96	341	377 [M+Cl]^−^, 179, 135	caffeoyl-*O*-hexoside	+	+	[108]
**3**	0.96	315	153, 152, 109, 108	dihydroxybenzoyl-*O*-hexoside	+	+	[94,109]
**4**	1.00	353	191, 179, 135	3-*O*-caffeoylquinic acid	+	+	[94,110,111]
**5**	1.02	417	405, 285, 223, 152	dihydroxybenzoyl-di-*O*-pentoside ^d^	+	+	[112,113]
**6**	1.15	357	195	caffeoyl-*O*-pentahydroxyhexanoic acid	-	+	[94]
**7**	1.23	305	225, 167, 147, 135	epigallocatechin/gallocatechin isomer ^d^	+	+	[114]
**8**	1.25	563	387, 327, 297, 223, 207, 205	dihydrosinapoyl-*O*-hexuronosyl-hexoside ^d^	+	+	[113]
**9**	1.29	353	707 [2M−H]^−^, 191	*trans*-5-*O*-caffeoylquinic acid	+	+	[94,110,111]
**10**	1.30	307	227, 189, 167, 148	catechin/epicatechin hydrated ^d^	+	-	[114,115]
**11**	1.35	353	191, 179, 173	4-*O*-caffeoylquinic acid	-	+	[94,110,111]
**12**	1.41	325	651 [2M−H]^−^, 163, 119	*p*-coumaroyl-*O*-hexoside isomer	-	+	[108]
**13**	1.55	593	1187 [2M−H]^−^, 503, 473, 383, 353	apigenin-6,8-di-*C*-hexoside	+	+	[94,110,116,117]
**14**	1.70	353	191	*cis*-5-*O*-caffeoylquinic acid	-	+	[94,110,111]
**15**	1.74	637	351, 285	luteolin-di-*O*-hexuronoside ^d^	+	+	[118]
**16**	1.78	387	775 [2M−H]^−^, 207, 163, 119	dihydrosinapoyl-*O*-hexoside ^d^	+	+	[113,119]
**17**	1.86	339	179, 161, 135	caffeoyl-*O*-dimethyl-dihydroxybutanedioic acid ^d^	+	+	[113]
**18**	1.97	535	517, 373, 341, 323, 281, 251, 209, 193, 179, 161, 149, 135	hydroxypinoresinol-*O*-hexoside ^d^	+	+	[113]
**19**	2.01	389	345, 227, 209, 183, 165	oleoside ^d^/secologanside ^d^	+	+	[120,121]
**20**	2.23	371	249, 121	unknown	+	+	-
**21**	2.28	563	503, 473, 443, 413, 383, 353	apigenin-8-*C*-hexosyl-6-*C*-pentoside ^d^	+	+	[117,122,123]
**22**	2.30	367	191	5-*O*-feruloylquinic acid	+	+	[108,111]
**23**	2.34	623	1247 [2M−H]^−^, 605, 561, 447, 327, 285	luteolin-*O*-hexuronosyl-hexoside	+	+	[94,113]
**24**	2.58	325	163, 119	*p*-coumaroyl-*O*-hexoside isomer	+	+	[108,124]
**25**	2.60	343	167, 113	vanillyl-*O*-hexuronoside ^d^	-	+	[125,126]
**26**	2.63	367	193, 179, 134	3-*O*-feruloyl-quinic acid	+	+	[111,127]
**27**	2.72	429	475 [M+HCOO]^−^, 411, 257, 227	unknown	+	+	-
**28**	2.84	799	619, 513, 351, 285, 193, 179, 161, 135	luteolin-*O*-hexuronosyl-caffeoylhexuronoside ^d^	-	+	[128]
**29**	2.90	393	378, 355, 319, 295, 283	unknown	+	+	-
**30**	3.05	829	414 [M−2H]^2−^, 649, 513, 351, 315, 300, 179, 161, 135	tetrahydroxy-methoxyflavone-*O*-hexuronosyl-caffeoylhexuronoside ^d^	-	+	[113,129]
**31**	3.15	651	299	trihydroxy-methoxyflavone-*O*-hexuronosyl-hexuronoside ^d^	+	+	[130]
**32**	3.20	681	329	trihydroxy-dimethoxyflavone-*O*-hexuronosyl-hexuronoside ^d^	+	+	[130]
**33**	3.54	431	341, 311, 283, 269	apigenin-8-*C*-hexoside ^d^	+	+	[110,131,132]
**34**	3.70	677	515, 353, 341, 323, 191, 179, 161	3,5-*O*-dicaffeoylquinic acid-*O*-hexoside	-	+	[94,111,133]
**35**	3.85	609	300, 301, 271, 255	rutin ^c^	+	+	[12,94,134,135]
**36**	3.90	607	269	apigenin-*O*-hexuronosyl-hexoside ^d^	+	+	[136,137]
**37**	4.07	461	923 [2M−H]^−^, 405, 285	luteolin-*O*-hexuronoside	+	+	[94,135,138]
**38**	4.17	593	417, 285	luteolin-*O*-hexuronosyl-pentoside ^d^	-	+	[12,94,138,139]
**39**	4.23	491	983 [2M−H]^−^, 315, 287	tetrahydroxy-methoxyflavone-*O*-hexuronoside	+	+	[94,135]
**40**	4.30	637	1275 [2M−H]^−^, 299, 284	trihydroxy-methoxyflavone-*O*-hexuronosyl-hexoside ^d^	+	+	[137]
**41**	4.31	667	1335 [2M−H]^−^, 491, 329, 314, 300	trihydroxy-dimethoxyflavone-*O*-hexuronosyl-hexoside ^d^	+	+	[130,137]
**42**	4.39	463	301, 300	quercetin-3-*O*-hexoside	-	+	[12,94,134,139]
**43**	4.46	785	623, 461, 285, 179, 161	luteolin-*O*-hexuronosyl-caffeoylhexoside ^d^	-	+	[94,113,135]
**44**	4.57	665	285	luteolin-*O*-hexuronosyl-acetylhexoside ^d^	+	+	[113]
**45**	4.82	477	315, 300	tetrahydroxy-methoxyflavone-*O*-hexoside	-	+	[94,135]
**46**	4.94	813	329, 314, 299, 163, 145	trihydroxy-dimethoxyflavone-*O*-hexuronosyl-coumaroylhexoside ^d^	-	+	[113,130]
**47**	5.14	445	313	dihydroxy-dimethoxyflavone-*O*-pentoside ^d^	+	+	[140]
**48**	5.28	843	865 [M−2H+Na]^−^, 681, 351, 329, 314, 299, 193, 179, 161, 135	trihydroxy-dimethoxyflavone-*O*-feruloyl-caffeoylhexuronoside ^d^	+	+	[113,130]
**49**	5.48	515	353, 335, 191, 179, 173, 161, 135	3,4-*O*-dicaffeoylquinic acid	-	+	[94,109,133,135,141,142]
**50**	5.95	515	353, 191, 179, 135	3,5-*O*-dicaffeoylquinic acid	-	+	[94,109,133,135,141,142]
**51**	6.45	515	353, 335, 191, 179, 135	1,3-*O*-dicaffeoylquinic acid ^d^	-	+	[109,133,141]
**52**	6.48	445	269	apigenin-*O*-hexuronoside	+	+	[94]
**53**	6.55	799	315, 300, 163, 145	tetrahydroxy-methoxyflavone-*O*-hexuronosyl-coumaroylhexoside ^d^	-	+	[113,137]
**54**	6.60	829	329, 314, 299, 179, 161, 135	trihydroxy-dimethoxyflavone-*O*-hexuronosyl-caffeoylhexoside ^d^	-	+	[113,137]
**55**	6.80	471	429, 411, 399, 267, 152	unknown	+	-	[113]
**56**	6.85	507	345, 330, 315, 287	tetrahydroxy-dimethoxyflavone-*O*-hexoside ^d^	-	+	[140]
**57**	7.01	769	635, 623, 593, 327, 285, 163, 145	luteolin-*O*-hexuronosyl-coumaroylhexoside ^d^	+	+	[113]
**58**	7.25	505	329, 314, 299, 271	trihydroxy-dimethoxyflavone-*O*-hexuronoside	+	+	[94,130,135]
**59**	7.43	503	445, 285, 175	luteolin-*O*-acetylhexuronoside ^d^	+	-	[143]
**60**	7.50	507	329, 314, 299, 285, 271	tetrahydroxy-dimethoxyflavone-*O*-hexoside ^d^	-	+	[140]
**61**	7.51	503	285, 175	luteolin-*O*-acetylhexuronoside ^d^	-	+	[143]
**62**	7.65	561	369, 351, 191	caffeoylquinic acid derivative ^d^	+	-	[109]
**63**	7.86	515	353, 317, 191, 179, 173, 135	4,5-*O*-dicaffeoylquinic acid	-	+	[94,109,133,135,141,142]
**64**	8.05	679	379, 299, 284	trihydroxy-methoxyflavone-*O*-hexuronosyl-acetylhexoside ^d^	+	+	[140]
**65**	8.25	501	367, 227, 193, 191, 179, 173, 161, 134	3-*O*-feruloylquinic acid derivative ^d^	-	+	[111,127]
**66**	8.73	521	399, 152	trihydroxydihydrochalcone-di-*C*-pentoside ^d^	+	+	[113]
**67**	8.88	417	285	luteolin-*O*-pentoside ^d^	-	+	[143]
**68**	8.94	501	537 [M+Cl]^−^, 399, 351, 152, 137	trihydroxydihydrochalcone-*C*-glycoside derivative ^d^	+	+	[113]
**69**	9.24	445	491 [M+HCOO]^−^, 313	dihydroxy-dimethoxyflavone-*O*-pentoside ^d^	+	+	[94,109,135,140]
**70**	9.69	813	667, 329, 314, 299, 285, 163, 145	trihydroxy-dimethoxyflavone-*O*-hexuronosyl-coumaroylhexoside ^d^	+	+	[140]
**71**	9.70	461	323, 299, 179, 161, 137	salicylic acid caffeoyl-*O*-hexoside ^d^	-	+	[135]
**72**	10.18	487	383, 353, 323, 285, 179, 163, 161, 145, 119	coumaroyl-caffeoyl-*O*-hexoside ^d^	+	+	[135]
**73**	10.34	549	387, 207, 179, 161	medioresinol-*O*-hexoside ^d^/eucommin A ^d^/dihydrosinapoyl-caffeoyl-*O*-hexoside ^d^	+	+	[109,113,144,145]
**74**	10.48	753	607, 269, 163, 145	apigenin-*O*-hexuronosyl-coumaroylhexoside ^d^	+	+	[113]
**75**	10.58	783	637, 299, 284, 163, 145	trihydroxy-methoxyflavone-*O*-hexuronosyl coumaroyhexoside ^d^	+	+	[113]
**76**	10.94	913	935 [M-2H+Na]^−^, 811, 769, 327, 285, 205, 175, 163, 145, 119	luteolin-*O*-coumaroylglycoside derivative ^d^	+	+	[143]
**77**	11.14	547	583 [M+Cl]^−^, 507, 487, 329, 314, 299	trihydroxy-dimethoxyflavone derivative ^d^	+	+	[94,135,140]
**78**	11.73	445	481 [M+Cl]^−^, 313	dihydroxy-dimethoxyflavone-*O*-pentoside ^d^	+	+	[94,135,140]
**79**	12.46	419	153, 152, 121, 109, 108	dihydroxybenzoyl-benzoyl-*O*-hexoside ^d^	+	+	[143]
**80**	12.76	783	367, 337, 299, 284, 269, 205, 163, 145	trihydroxy-methoxyflavone-*O*-coumaroyl-sinapoylhexoside ^d^	+	+	[143]
**81**	12.83	285	257	luteolin ^c^	+	+	[12,94,109,135,138,139,140,146,147]
**82**	13.01	315	300	dihydroxy-methoxyflavone	+	+	[94,140]
**83**	13.12	345	330, 315, 300, 287, 271	tetrahydroxy-dimethoxyflavone	+	+	[94,140]
**84**	13.36	677	515, 353, 191, 179, 173	3,4,5-tri-*O*-caffeoylquinic acid	+	+	[94,111,133,148,149]
**85**	13.62	345	330, 315, 300, 287, 271	tetrahydroxy-dimethoxyflavone	-	+	[94,140]
**86**	13.74	269	227	apigenin ^c^	+	+	[94,140]
**87**	13.98	299	-	trihydroxy-methoxyflavone	+	+	[94,140]
**88**	14.04	359	344, 329, 327, 314, 299, 284	trihydroxy-trimethoxyflavone	+	+	[94,140]
**89**	14.07	329	314, 299, 284, 271	trihydroxy-dimethoxyflavone	+	+	[94,140]
**90**	14.34	359	344, 329, 314, 299, 285, 271	trihydroxy-trimethoxyflavone ^d^	+	+	[94,140]
**91**	14.42	359	344, 329, 314, 301, 286	trihydroxy-trimethoxyflavone	+	+	[94,140]
**92**	14.87	343	328, 313, 298	dihydroxy-trimethoxyflavone	+	+	[94,140]

^a^ Compound numbers and retention times (t_R_) refer to UV chromatograms shown in Figure 2A,B; ^b^ Abbreviations: TbE: *T. balsamita* 50% (*v/v*) ethanolic extract; TbW: *T. balsamita* aqueous extract; +: present in the extract; -: not present in the extract; ^c^ Compared to a reference standard; ^d^ Reported for the first time in *T. balsamita*.

**Table 2:** Results of the PAMPA experiments expressed as log*P_e_* values (n = 9).

Compound	log*P_e_* PAMPA-BBB (n = 9)	log*P_e_* PAMPA-GI (n = 9)
Tetrahydroxy-dimethoxyflavone (**85**)	−4.78 ± 0.15	−4.54 ± 0.13
Apigenin (**86**)	−4.46 ± 0.11	−4.75 ± 0.26
Trihydroxy-methoxyflavone (**87**)	−4.56 ± 0.17	−4.56 ± 0.19
Trihydroxy-trimethoxyflavone (**88**)	−4.88 ± 0.13	−4.75 ± 0.18
Trihydroxy-dimethoxyflavone (**89**)	−4.46 ± 0.14	−4.53 ± 0.13
Trihydroxy-trimethoxyflavone (**90**)	−4.54 ± 0.08	−4.74 ± 0.13
Trihydroxy-trimethoxyflavone (**91**)	−4.43 ± 0.21	−4.74 ± 0.14
Dihydroxy-trimethoxyflavone (**92**)	−4.30 ± 0.13	−4.93 ± 0.13

## Data Availability

The data presented in this study are available on request from the corresponding author.
